# Good news on the active management of pregnant cancer patients

**DOI:** 10.12688/f1000research.22472.1

**Published:** 2020-06-01

**Authors:** Susan M. Folsom, Teresa K. Woodruff

**Affiliations:** 1 Department of Obstetrics and Gynecology, Northwestern University, 250 East Superior Street, Suite 03-2303, Chicago, IL, 60611, USA

**Keywords:** Cancer, Pregnancy, Chemotherapy, Radiation, Surgery

## Abstract

Cancer occurs in approximately 1/1000 to 1/2000 pregnancies and presents complex medical and ethical dilemmas for patients and providers. The most common cancers diagnosed in the gestational period include breast, cervical, melanoma, and lymphomas. The majority of existing evidence regarding the treatment of cancer during pregnancy is derived from experiences with breast cancer. Other cancers often pose unique challenges given the location of the tumors and their traditional mode of treatment with pelvic surgery and radiation. Additionally, many emerging therapies for cancer target mechanisms that are necessary for fetal development, such as angiogenesis, and are contraindicated in pregnant women. Although limitations on the treatment of cancer during pregnancy currently exist, increasing evidence shows that many surgical and systemic therapies can be effective for a mother’s oncologic outcomes without significant detriment to the developing fetus. Traditional perspectives of cancer during gestation may sway providers to encourage pregnancy termination, delays in therapy, or early delivery. However, recent studies and reviews discourage such practices. Although every cancer diagnosis in pregnancy requires an individualized approach and should use the multidisciplinary perspectives of maternal–fetal medicine specialists as well as medical and surgical oncologists, providers should feel empowered to safely employ systemic, surgical, and even reserved cases of radiation therapies for their pregnant patients with cancer. The aim of this review is to highlight some of the recent advances in cancer therapies for common cancer subtypes and encourage providers to use this growing body of evidence to employ treatments with curative intent while continuing to evaluate the long-term effects of these therapies on mothers and their children.

## Introduction

Cancer in pregnancy is rare, occurring in approximately 1/1000 to 1/2000 gestations ^1^. In many publications, gestational cancer is defined as cancer until the first year postpartum ^2^. Publications from the International Network on Cancer, Infertility and Pregnancy (INCIP), based in Europe, focus primarily on the diagnosis and treatment of cancer during the gestational period only. Given that age is an independent risk factor for malignancy, the incidence of cancer during pregnancy is increasing as more women delay childbearing ^3^. With the advent of noninvasive prenatal testing (NIPT), there is also an increase in early diagnosis of cancer in pregnancy ^4^. With this increase in incidence, providers should gain both an understanding of and comfort with the diagnosis and treatment of cancer in pregnancy.

## Diagnosis

Diagnosing cancer in pregnancy can pose a significant challenge for patients and providers. During pregnancy, women may experience fatigue, constipation, abdominal discomfort, nausea, textural breast changes, and other symptoms that can confound the early diagnosis of cancer. Moreover, concerns regarding the exposure of a developing fetus to anesthesia, ionizing radiation, or teratogenic contrast agents can lead providers to pause or use substandard modalities to diagnose or stage a malignancy. X-ray, mammography, and even computed tomography and positron emission tomography have been used when clinically necessary to diagnose or stage a malignancy during pregnancy ^5, 6^. However, non-irradiating diagnostic modalities, including ultrasound (US) and magnetic resonance imaging (MRI) without gadolinium contrast, continue to be the recommended standard of care ^6^. As non-irradiating modalities continue to evolve, the need for irradiating studies will likely decrease. In a study published in 2018, Han *et al*. showed that whole-body diffusion-weighted imaging and MRI improves diagnostic assessments of gestational cancers ^7^. This imaging modality may replace traditional imaging studies used for cancer staging during pregnancy, including MRI and US ^7^. Tumor markers, such as CA-125 and CEA, are also important in the diagnosis of many malignancies. However, these markers are often altered during gestation, and there are few studies evaluating these tumor markers in pregnancy. An understanding of the validity of these markers during active fetal-placental development is lacking ^8^.

An exciting new development in terms of cancer diagnostics in pregnant women is the potential to use NIPT with cell-free DNA to detect pre-symptomatic malignancies. NIPT allows providers to screen fetal DNA in maternal blood for fetal trisomies and other chromosomal aneuploidies. Given the ability to look for aberrant chromosomes, researchers have found that they can use similar testing to screen maternal blood for aneuploidies characteristic of malignancy. In a case series by Amant *et al*., ovarian carcinoma, follicular lymphoma, and Hodgkin’s lymphoma were all discovered using a large, parallel sequencing–based dataset and analysis ^4^. This method not only evaluated cell-free DNA for common trisomies but also allowed the genome-wide discrimination of maternal and fetal DNA for copy-number variation characteristic of malignancy ^4^. Although this diagnostic method has been studied only on a small scale, it holds the potential for early, asymptomatic cancer diagnosis in pregnancy and beyond.

In summary, cancer in pregnancy can be difficult to diagnose, even by the most astute physician. Once there is a suspicion of malignancy, physicians should not hesitate to proceed with the imaging and biopsies necessary to confirm a diagnosis. Using methods such as NIPT may be a step toward recognizing malignancies at their earliest stages.

## Chemotherapy

Cancer is traditionally treated with three modalities: surgery, systemic therapy (including chemotherapy and other targeted therapies), and radiation. Delaying treatment in cancer worsens prognosis, but pregnancy presents unique challenges that may necessitate delays. A brief review of the effects of therapies on a pregnancy at various gestational ages is presented in Table 1. In the first trimester of pregnancy, all systemic chemotherapies can have teratogenic effects and should be avoided ^3, 5, 9– 11^. For cancers diagnosed near term, it may be prudent to delay treatment until after delivery if the period between diagnosis and delivery is only a matter of weeks. Despite increasing evidence in favor of good oncologic and pregnancy outcomes, many gynecologists may still favor termination or iatrogenic prematurity in lieu of therapy during a pregnancy ^12^.

**Table 1.** Summary of cancer therapies and their effects on gestation.

	Early first trimester conception-4 weeks	First trimester 4 weeks-14 weeks	Second trimester 14 weeks-28 weeks	Third Trimester 28 weeks-delivery
	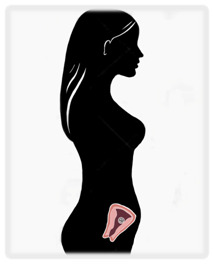	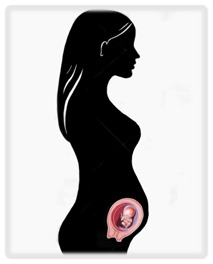	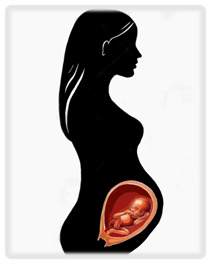	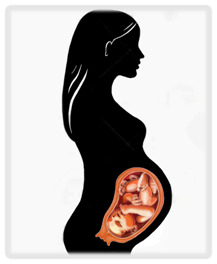
**Chemotherapy**	All or none ^a^	High risk of severe fetal malformation. Increased risk of miscarriage ^3, 8, 16, 17^	Growth restriction, low birth weight, preterm labor, myelosuppression, need for NICU admission ^3, 8, 17^	Growth restriction, low birth weight, preterm labor, myelosuppression, need for NICU admission ^3, 8, 17^
**Anti-HER2** **agents** ^b^	Fetus unaffected in review of limited case reports ^18^	Fetus unaffected in review of limited case reports ^18^	Oligohydramnios/ Anhydramnios ^18^	Oligohydramnios/ Anhydramnios ^18^
**Hormonal** **therapy** ^c^	Possible increased risk of miscarriage ^19^	Facial malformations, ambiguous genitalia, possible increased risk of miscarriage, some cases with no adverse effects observed, data limited to animal studies and case reports ^2, 19^	Insufficient data ^19^	Insufficient data ^19^
**Immunotherapies**	Increased risk of miscarriage ^3^	Increased risk of miscarriage ^3^	Increased risk of stillbirth, premature delivery, infant mortality ^3^	Increased risk of stillbirth, premature delivery, infant mortality ^3^
**Angiogenesis** **inhibitors**	All or nonee	Increased risk of miscarriage, skeletal malformations, abnormal vascular development of the skin, pancreas, kidney, and lung ^11^	Intrauterine growth restriction, preeclampsia, hypertension ^11^	Intrauterine growth restriction, preeclampsia, hypertension ^11^
**Radiation** **therapy** ^d^	All or none ^e^	Gross malformation, small head size, mental retardation ^20^	Mental and growth retardation, small head size, cataracts, sterility, secondary malignancies ^20^	Growth retardation, sterility, cataracts, secondary malignancies ^20^
**Surgery** ^e^	1–2% increased risk of miscarriage ^8^	1–2% increased risk of miscarriage ^8^	Premature delivery ^8^	Premature delivery ^8^

^a^An “all or none” effect means that the pregnancy either will fail or will continue without issue. ^b^These effects are derived largely from studies of trastuzumab. ^c^Effects listed are those seen in studies evaluating tamoxifen use in pregnancy. ^d^Effects seen at fetal exposures of more than 0.3 Gy ^9^. ^e^Risks seen primarily in abdominal and pelvic surgery. NICU, neonatal intensive care unit. Illustrations by Katelyn Folsom.

Chemotherapy during pregnancy carries risks that depend primarily on the therapy’s mechanism of action. Many chemotherapies involve restricting the growth of rapidly proliferating cells and other anti-metabolite properties. Although evidence continues to support the recommendation against chemotherapy during organogenesis and the first trimester of pregnancy, many chemotherapeutic agents, when used in the second and third trimesters, have shown favorable safety profiles for mothers and fetuses ^3, 5, 9– 11^. Chemotherapy should be discontinued 3 to 5 weeks prior to anticipated delivery in order for maternal bone marrow to recover and chemotherapeutic agents to be cleared from the fetal circulation ^5, 6, 9, 10^. As use of these therapies has increased, so has evidence of good neonatal and pediatric outcomes ^13^.

The physiology of pregnancy, including but not limited to changes in glomerular filtration rate, anti-diuretic hormone secretion, hepatic metabolism, and acid–base balance, may affect the metabolism and action of systemic therapies in pregnant women. Studies evaluating the efficacy of systemic therapies in the context of maternal physiologic changes are limited but show decreased dose exposure in pregnant compared with non-pregnant women ^14^. Although dose adjustments for pregnant cancer patients is an active area of study, current recommendations endorse weight-based dosing, similar to chemotherapeutic dosing for non-pregnant patients. This dosing should be adjusted with maternal weight changes throughout pregnancy and the postpartum period ^5, 6, 8^.

## Surgery

Surgery and anesthesia have been shown to be safe in pregnancy despite a slightly increased risk of low birth weight, preterm labor, and miscarriage (1–2%) ^8, 15^. This risk is primarily increased for patients who receive major abdominal or pelvic surgeries ^8^. Depending on the type of cancer, most surgeries should be carried out during the early second trimester to minimize the risks of anesthesia to the fetus during organogenesis while operating before the gravid uterus obstructs visualization of the target anatomy ^15^. Loco-regional anesthesia is preferred, and women carrying gestations greater than 20 weeks should be positioned with a leftward or rightward tilt to alleviate the effects of the gravid uterus on the inferior vena cava ^15^. Fertility preservation, often a priority for women with cancer who are of reproductive age, should be a part of a patient’s counseling and surgical planning ^21^. Fertility preservation may assist in coping with a cancer diagnosis and should involve the expertise of oncofertility specialists ^22^. This desire to preserve fertility may alter surgical management of gynecologic and gastrointestinal malignancies in pregnant patients ^23^. This is discussed below in the “Ovarian cancer” and “Cervical cancer” sections. Despite these limitations, it is important to consider the urgency one would feel while choosing to operate on a non-pregnant patient. Although there are obvious exceptions (such as gynecologic cancers involving the uterus), many pregnant women should receive the same surgical care as their non-pregnant counterparts.

## Radiation therapy

Radiation carries particular challenges and is presumed to have deleterious effects on fetal outcomes throughout gestation ^5, 11^. In most cases, radiation therapy has been considered an absolute contraindication in pregnancy. However, as stereotactic radiation methods and improved modalities of administration are developed, radiation therapy may be a possibility for more women during pregnancy ^8, 24^. The threshold for adverse radiation effects in fetuses is less than 100 mGy ^16^. Via methods that isolate areas affected by certain cancer subtypes, such as melanoma, Hodgkin’s lymphoma, and even breast cancer, the amount of fetal radiation exposure can be reduced. This may allow the possibility of radiation therapy without disrupting or negatively affecting a pregnancy, depending on gestational age and the distance of the field of irradiation to the fetus ^24^.

## Breast cancer

Breast cancer, which has an incidence of 15 to 35 per 100,000 pregnancies, is both the most frequent and also the most well-studied cancer in pregnancy ^3^. Breast cancer is difficult to diagnose in the gestational and postpartum period. During pregnancy, breasts undergo changes in texture and structure, including increased breast density and increased water content ^16^. Given these changes, if there is a palpable mass, there should be a low threshold for mammographic evaluation and subsequent core needle biopsy in a pregnant woman ^9^. The histologic subtypes of breast cancer that are detected in pregnancy are typically poorly differentiated invasive ductal carcinoma which are estrogen/progesterone receptor–negative. Pregnant women tend to have HER2 positivity at rates similar to those of the general population ^5, 6, 25^.

Perhaps somewhat unexpected to providers is the evidence that therapies traditionally used in breast cancer, such as anthracyclines, cyclophosphamide, and taxanes, can all be used in pregnant women after the first trimester ^6^. When these therapies are used in the second and third trimesters, some studies have shown intrauterine growth restriction, prematurity, low birth weight, and myelosuppression. However, the rate of these adverse effects is about 3%, the same rate as that of the general population ^3^. In 2015, Amant *et al*. published a multi-center, prospective, case-control study involving 129 children exposed to cancer and cancer therapy during gestation and compared outcomes with their matched controls ^13^. That study confirmed what had been seen in other, smaller case series: the effects of prematurity more significantly impacted child growth and development than the effects of chemotherapy ^13^. The subjects iatrogenically born prematurely had outcomes similar to premature, matched controls, regardless of the fetus’ exposure to cancer therapy ^6, 11^. Termination of pregnancy and iatrogenic premature delivery in order to deliver chemotherapy for breast cancer are not supported by current evidence ^6, 9, 25^.

With regard to maternal outcomes, some small studies have shown a poorer prognosis for women with breast cancer diagnosed in pregnancy ^26^. However, in the largest cohort study following maternal outcomes in pregnancy-associated breast cancer to date, pregnant women who receive treatment for breast cancer had disease-free survival and overall survival comparable to those of the general population ^27^. Patients should receive treatment with curative intent and should avoid delays as much as possible.

Hormonal and targeted therapies are largely contraindicated in pregnancy. Targeted therapies, such as trastuzumab, have been associated with poor fetal outcomes, including oligohydramnios, which can lead to pulmonary hypoplasia, developmental anomalies, and ultimately fetal death ^3, 28^. Tamoxifen is also contraindicated because of increased reports of malformations in fetuses exposed to tamoxifen. However, much of this exposure took place in the first trimester, and information regarding exposure during the second and third trimesters is lacking ^19^. Ultimately, with regard to targeted therapies, more data are needed to make appropriate recommendations regarding their use, and large registries through maternal–fetal medicine and oncology practices nationwide would be critical to developing ongoing guidance. Until more information is available, use of these agents in pregnancy should be avoided.

The surgical management of breast cancer poses minimal fetal risk. In the past, mastectomy with axillary lymphadenectomy (depending on the stage of the cancer) was recommended as the standard treatment for breast cancer in pregnancy. However, breast-conserving surgery is emerging as a viable option for many patients with early-stage disease ^3^. Recent studies have shown sentinel lymph node dissection to be effective and have minimal adverse effects on the fetus ^24, 29^.

Fetuses may be exposed to radiation from a variety of sources, and it is important to consider the difference between fetal and maternal exposure. The threshold for deleterious radiation effects in fetuses is less than 100 mGy ^16^. Chest wall radiotherapy for breast cancer exposes the mother to 40 to 60 Gy of radiation, and fetal exposure is as high as 2 Gy. Given this high dosage of fetal radiation, radiation therapy for breast cancer in pregnancy is still considered an absolute contraindication ^3, 5, 6, 25^, although this may change in coming years with improving technologies. Until then, women who opt for breast conservation should receive adjuvant chemotherapy followed by radiation therapy postpartum ^16^.

## Hodgkin’s lymphoma and hematologic malignancies in pregnancy

Hodgkin’s lymphoma, which occurs in about 5 to 8 in 100,000 pregnancies, is the most common hematologic malignancy in pregnancy ^30^. Given the aggressive nature of hematologic diseases and their responsiveness to chemotherapy, it is particularly important that delays in diagnosis and treatment do not occur for these patients ^31^. Patients should undergo chemotherapy with the standard treatment regimen—doxorubicin, bleomycin, vinblastine, and dacarbazine (ABVD)—after completion of the first trimester. This regimen has a safety profile similar to that of other chemotherapies in the second and third trimesters. In a multi-center retrospective cohort study published in 2019 ^30^, patients who received ABVD had an increased incidence of preterm contractions and preterm premature rupture of membranes but did not show the increased percentage of small-for-gestational-age infants which was seen in studies of infants exposed to taxanes and platinum-based chemotherapy ^32^. Although radiation is considered a mainstay of therapy for patients with Hodgkin’s lymphoma, current evidence supports additional chemotherapy cycles until radiation therapy can be pursued in the postpartum period. For patients with lesions causing spinal cord compression, superior vena cava syndrome, or large mediastinal masses, palliative radiation with measures to avoid fetal exposure may be acceptable while awaiting fetal maturity and delivery ^31, 33^.

Though far more rare, other hematologic malignancies, including acute leukemias, require even more timely treatment. If diagnosed during the first trimester, acute leukemia may be a malignancy that cannot afford a treatment delay until the second trimester. Acute leukemias are highly chemosensitive, and chemotherapy delayed by even one week for fetal benefit has shown worse maternal outcomes ^34^. Counseling patients toward termination may be appropriate if acute myeloid leukemia, acute promyelocytic leukemia, or acute lymphocytic leukemia is diagnosed in the first trimester ^34^.

## Cervical cancer

Cervical cancer is the most common gynecologic malignancy in gestation but is still a rare occurrence. Reported incidences range from 1.4 to 4.6 per 100,000 pregnancies ^10^. Given its location and the fact that surgical treatment is preferred for early-stage disease, the treatment of cervical cancer in pregnancy is particularly challenging. If the tumor is diagnosed at an early stage (IA1 to IB1 tumors), fertility-sparing surgery may be an option. Although the standard of care outside of pregnancy for these tumors remains hysterectomy, recent studies have explored the use of a cold knife conization or a loop electrosurgical excision procedure with a top hat and subsequent McDonald cerclage ^10^. In a matched cohort study published in 2019, Halaska *et al*. showed that outcomes for pregnant women who underwent surgery and chemotherapy were similar to those of their matched non-pregnant counterparts ^35^. Careful counselling about the high risk of miscarriage or preterm birth with these procedures should also take place; however, in the cohort of Halaska *et al*., the six patients who underwent surgical conization in pregnancy delivered at an average gestational age of 37 weeks. Similar outcomes have been reported in other studies ^35^. For more advanced stages of cervical cancer, systemic therapy with traditional platinum-based therapies with or without paclitaxel should be used. A recent international cohort study analyzing 1170 cases of cancer in pregnancy showed higher rates of small-for-gestational-age infants with platinum- and taxane-based chemotherapies ^32^; however, other data have shown no effect on long-term outcomes in infants exposed to such chemotherapy ^13^. Radiation therapy is contraindicated until the postpartum period given the location of cervical tumors.

The route of delivery should be carefully considered on the basis of the size of the cervical tumor. If disrupted during a vaginal delivery, large bulky tumors may predispose patients to significant hemorrhage. For such tumors, Cesarean delivery is recommended ^36^. Although previous studies supported vaginal delivery for patients with diagnosed microinvasive disease, reports of fatal recurrences of cervical cancer at the episiotomy site have led experts in the field to recommend Cesarean delivery regardless of tumor size ^37^. A radical hysterectomy, if indicated by the size of tumor, should be performed at the time of delivery. Providers should prepare for the increased surgical complexity of the postpartum uterus and anticipate increased blood loss ^35, 36^.

## Melanoma

Melanoma is one of the most frequent malignancies encountered during gestation and is common in younger women. It occurs in approximately 2.8 to 5.0 per 100,000 pregnancies, and about 35% of women with melanoma are of childbearing age. The prognosis of melanoma is not altered significantly by pregnancy, but the outcomes for the mother and fetus are highly dependent on the stage of the disease ^38^.

Surgical management of melanoma should not be altered in pregnancy ^24, 38^. Depending on the stage of the tumor, lymphadenectomy or sentinel lymph node biopsy may be necessary. In the literature of both melanoma and breast cancer, the use of isosulfan blue dye continues to be discouraged because of its potential for anaphylactic reactions. Its substitute, methylene blue, is discouraged due to its teratogenic effects in the first trimester ^35^. Sentinel lymph node dissection with technetium 99 is controversial but is currently thought to have acceptably low levels of fetal exposure to radiation ^38, 39^. Ultimately, surgical management of melanoma is considered safe.

Increasing evidence also exists regarding the safe use of radiotherapy in selected cases of melanoma. Modalities such as stereotactic ablative radiation therapy and intensity-modulated radiation may allow irradiation of malignancies remote from the maternal pelvis without significantly affecting the fetus ^24^. Gamma knife therapy also has been reportedly used safely in pregnancy but data are still scant in this regard ^24^.

Although surgical excision, the standard for early-stage melanoma, is considered safe in pregnancy, advanced-stage disease presents significant challenges. Chemotherapy once was considered the only option for advanced-stage melanoma but since has been shown to be ineffective. In recent years, targeted therapies, including BRAF inhibitors, have been shown to be effective in advanced-stage disease and now are considered the standard of care ^39^. These therapies are still discouraged in pregnancy. Their safety profiles in human and animal models have shown significant teratogenic effects ^38^. In cases of stage III or IV melanoma, it may be necessary to deliver a patient prematurely or terminate a first-trimester pregnancy in order to optimize oncologic outcomes ^38^.

Interferon-alpha, used in patients with high-risk melanoma, is a safe treatment modality during pregnancy. With its high molecular weight, it does not cross the placenta. However, its significant side-effect profile may limit its use and lead to discontinuation of the therapy ^24^.

## Ovarian cancer

Although ovarian cancer itself is exceedingly rare during pregnancy (4 to 8 per 100,000 pregnancies), adnexal masses are a common finding in pregnant women. The majority of these adnexal masses are mature teratomas, followed by serous cystadenoma, mucinous cystadenoma, and endometrioma ^10^. Outside of pregnancy, epithelial ovarian cancer is the most common subtype and usually is seen in older women. During pregnancy, germ cell and sex cord stromal tumors are more frequent ^10^. There should be a low threshold for obstetricians and gynecologists to operate on large or suspicious adnexal masses ^40^. Although the chances of malignancy may be low, pregnant women are also at risk of torsion in the first and second trimesters ^41^. Surgical management of suspicious adnexal masses can prevent torsion and also provide a definitive diagnosis. If cancer is diagnosed, fertility-sparing staging can be pursued with lymphadenectomy, peritoneal and omental biopsies, and appendectomy as indicated. For more advanced stages of ovarian cancer or for disease that is deemed unresectable, neoadjuvant chemotherapy should be given, just as in the non-pregnant population. A platinum derivative combined with paclitaxel is thought to have a reasonable safety profile in pregnancy, as discussed above. For non-epithelial ovarian cancer, the standard chemotherapy is bleomycin, etoposide, and cisplatin (BEP). Given the toxicities reported in etoposide, oncologists may consider alternative chemotherapeutic regimens such as cisplatin, vinblastine, and bleomycin (PVB) ^28^.

## Conclusions

Cancer in pregnancy, though increasing in incidence, is still a rare occurrence. Given its rarity, evidence has long been based on animal studies, small case reports, and case series. However, in recent decades, a growing number of studies evaluating pregnant patients with cancer treated with systemic therapy have confirmed the short-term safety of many therapies and their use in pregnancy. As evidence has accrued, it has supported the use of many systemic therapies and surgical treatments for various cancer subtypes without interrupting gestation. However, there are still major gaps in our knowledge regarding the long-term effects on fetuses and maternal outcomes. Data are particularly limited in the realm of targeted therapies. Given that these therapies are now the standard treatment for many cancers, they warrant further time and research. Radiation therapy, with advancing techniques, allows more precise targeting of irradiated fields. This may be used more often in pregnant patients with cancer in the coming years. Surgery has long been considered safe in pregnancy, and surgeons with experience operating on pregnant women should be employed to aid in the treatment of these rare diagnoses. In some instances, providers may feel that offering termination during early pregnancy is important for appropriate cancer care, but it should not be deemed the only option available for patients.

It is imperative to foster a multidisciplinary approach in the care and treatment for pregnant women with cancer. Although increasing evidence is supporting the use of safe and effective cancer therapies in pregnancy, the stage of the disease, age of gestation, and oncologic subtype make every case unique. An individualized approach should help shape the treatment plan for each patient. Medical teams should include maternal–fetal medicine specialists, oncologists, radiologists, and surgeons while paying special attention to patients and their individual wants and desires. As needed, the inclusion of ethics and palliative care teams may be helpful in navigating the challenging decisions that malignancy during gestation can present.
